# A decade of human uterus transplantation

**DOI:** 10.1111/aogs.15080

**Published:** 2025-02-19

**Authors:** Mats Brännström

**Affiliations:** ^1^ Department of Obstetrics and Gynecology, Institute of Clinical Sciences, Sahlgrenska Academy University of Gothenburg Gothenburg Sweden

On September 4, 2014, the birth of the first child from a transplanted uterus marked a monumental milestone in the fields of gynecology, reproductive medicine, and obstetrics.^1^ This historic achievement, realized in Sweden, established uterus transplantation (UTx) as a groundbreaking treatment for absolute uterine factor infertility—a condition affecting approximately 1 in 500 women. Since that pioneering UTx birth, over 70 children have been born globally following more than 130 UTx procedures.^2^ The world's first eight live births after UTx took place at Sahlgrenska University Hospital, Sweden.

On September 5, 2024, the International Society of Uterus Transplantation convened in Gothenburg for its 4th State‐of‐the‐Art Meeting. The event began with a heartwarming moment: the first UTx child born, who had celebrated his 10th birthday the day before, addressed the audience. Speaking in fluent English, he described himself as “an ordinary boy who loves sports, especially golf and ice hockey.” He shared how proud his parents were of him and encouraged the attendees to continue their work to make UTx widely accessible. His words deeply moved the delegates, many of whom shed tears of joy, reflecting on the remarkable progress made in just a decade and the profound human impact of this groundbreaking medical advancement within our medical field.

The UTx journey from concept to reality is widely regarded as a prime example of a translational project in innovative surgery, meticulously prepared and monitored according to the highest standards outlined in the Moore Criteria^3^ and the IDEAL framework^4^ for introducing major surgical innovations. As part of this rigorous approach, systematic animal research was conducted across several species,^5^ before the first clinical UTx‐trial started in 2012–2013.^6^

The field of UTx has evolved rapidly over the past decade, with continuous methodological advancements aimed at improving both the safety and efficacy of the procedure. The surgery itself, particularly live donor (LD) hysterectomy, is highly complex. For a LD‐UTx, the total surgical time—including hysterectomy, back‐table preparation, and transplantation—is around 15 h.^6^ To reduce tissue trauma, robotic surgery has been introduced for LD hysterectomy, leading to shorter hospital stays and reduced recovery time.^7^ However, this approach has not yet resulted in a reduction in overall surgical duration. In terms of immunosuppression (IS), the initial high‐IS induction regimen with antithymocyte globulin and high tacrolimus levels has now been adjusted to a moderate‐IS induction protocol using basiliximab and moderate tacrolimus levels. Our experience is that this modified IS protocol does not increase the risk of rejection episodes and has reduced the previously observed nephrotoxic effects of IS in UTx patients.^8^ For diagnosing rejection, the gold standard remains the histological evaluation of ectocervical biopsies, a method initially developed in a non‐human primate UTx model.^9^

This theme issue of AOGS contains 11 original research papers, one bibliographic analysis, and one systematic review, all with the common scientific goal of advancing the UTx field. The issue opens with a bibliometric analysis by Akbari and coworkers covering UTx research from 1960 to 2024, highlighting the growth of this expanding discipline.^10^ The origins of UTx research date back to the 1960s, initially focusing on utero‐tubal transplantation as a more feasible surgical approach compared to isolated tubal transplantation, to treat tubal factor infertility. Following more than a decade of preclinical studies, the groundbreaking birth of Louise Joy Brown in 1978—made possible through the pioneering work of the reproductive biologist Robert Edwards, the laparoscopist Patrick Steptoe, and the nurse/embryologist Jean Purdy—demonstrated that in vitro fertilization (IVF) was a viable solution for tubal infertility. The challenges faced by Edward's team, including skepticism from funding bodies, medical societies, and the media, are vividly depicted in the newly released and highly recommended Netflix film *Joy*. Interestingly, the path of UTx from concept to clinical reality^2^ shares many parallels with the pioneering journey of IVF.

While animal experimentation has been essential for the clinical introduction of UTx, continued research in this area remains crucial for the further advancement of human UTx. This theme issue includes four animal‐based studies. A new rat UTx model that preserves the bicornuate uterus shows promise for advancing research on UTx immunology, rejection, and maternal‐fetal tolerance during pregnancies in a uterine allograft, as outlined in an article by Polenz et al.^11^ Two studies investigate ways of decreasing ischemia of a uterine graft. In a pig model, Sousa et al. examine hypothermic machine perfusion as a means of prolonging uterine tolerance to cold ischemia.^12^ In the sheep model, Macedo Arantes and coworkers study sequential revascularization to decrease warm ischemia in UTx.^13^ Collectively, progress in this area will improve ischemic tolerance and reduce ischemia–reperfusion injury in human UTx. The theme issue also highlights animal research on uterus bioengineering, a potentially groundbreaking approach that could one day allow for customized uterus production, eliminating the challenges of organ shortages and rejection. This technique involves creating a uterus in a bioreactor, using patient‐specific stem cells. Previous studies in rodents have demonstrated success with bioengineered uterine tissue, and the current research study by De Miguel‐Gómez et al., exploring decellularization protocols in the baboon uterus, takes a step closer to human applications.^14^

In its early stages, UTx was viewed with skepticism as a potential infertility treatment due to concerns about the surgical risks, the side effects of IS for recipients, and possible impacts on fetal development and outcome of children. However, with the proven success of UTx resulting in live births across several countries,^2^ attitudes toward the procedure appear to have shifted in recent years. Two studies in this theme issue explore current perceptions of UTx among women with absolute uterine factor infertility, for whom gestational surrogacy (GS) would also be an option for achieving genetic motherhood, although GS is not practiced in these specific countries. The studies, conducted as a survey in Spain by Rius et al., and as an interview study in France, Sweden plus Norway by Carton et al. reveal high acceptance of UTx as a preferred method for achieving full biological motherhood, encompassing both genetic and gestational connections.^15^, ^16^

The majority of the more than 100 women worldwide who have undergone UTx to date have congenital uterine agenesis associated with Mayer‐Rokitansky‐Küster‐Hauser syndrome (MRKHs).^2^ Three papers in this theme issue explore the psychological aspects of women preparing for UTx, particularly those with MRKHs. Separate studies by Cospain et al. and Karpel et al. provide insights into the unique psychological challenges faced by women in France,^17^, ^18^ while Gerstl et al. offer an Australian perspective on women preparing for UTx.^19^ These studies highlight the importance of comprehensive psychological support, for women undergoing this complex, life‐changing procedure. On the same topic, Rabbani et al. from Birmingham, Alabama described their experience concerning the mental health of candidates in their large, deceased donor (DD) UTx program.^20^

Preparing to initiate a clinical study of human UTx at a new center should be a long‐term and systematic process.^2^ It is recommended that the first step involves establishing a multidisciplinary team at the new center. This team should then begin by conducting a thorough review of the key literature on human UTx, which currently includes fewer than 100 key papers. Collaboration with an experienced UTx center is essential during this preparatory phase. The new team should undertake surgical training in a large animal model, followed by an on‐site visit to observe UTx procedures at the experienced center.^2^ Finally, when the new center is ready to perform its first UTx surgeries, it is highly advisable to have experienced surgeons present to assist during these initial cases. The contribution by Tan et al. to this theme issue is a report by the collaborative Singapore‐Sweden team, detailing the transfer of expertise and the first surgically successful UTx case in Southeast Asia.^21^

A UTx procedure should not be considered fully successful until a healthy baby has been delivered from the transplanted uterus. Unlike classical solid organ transplants, where success can typically be assessed within a few days, UTx requires at least a year from transplantation to the confirmation of success through childbirth. Several case reports and case series on live births following UTx have been published, with the first birth from a LD‐UTx in 2014 and the first birth from a DD UTx in 2017, both documented in detailed articles in *The Lancet*, underscoring the significance of this emerging field.^1^, ^22^ A key contribution to this theme issue is a systematic review by the Gothenburg group of maternal and perinatal outcomes from 40 published pregnancies resulting in live births after UTx.^23^ The review highlights an increased risk of adverse obstetrical outcomes, emphasizing the need to manage these pregnancies as high‐risk.

In conclusion, a decade after the first successful UTx and subsequent birth, the UTx field has made remarkable progress in both clinical and research settings. The growing number of live births across the globe demonstrates that UTx is no longer an experimental concept but a viable fertility treatment for women with absolute uterine factor infertility. However, ongoing advancements are essential to refine surgical techniques, improve IS protocols, and minimize LD, maternal, and fetal risks. As this theme issue illustrates, collaboration between clinical teams and researchers will be crucial in overcoming remaining challenges and ensuring that UTx becomes a widely accessible and safe option for women seeking full biological motherhood.

## FUNDING INFORMATION

Jane and Dan Olsson Foundation for Science (2020–09 to MB), Swedish Research Council (2024–03487 to MB), Knut and Alice Wallenberg Foundation (2017.0363 to MB), and the Swedish state under the ALF agreement between the Swedish government and the county councils (ALFGBG‐965535 to MB).

## CONFLICT OF INTEREST STATEMENT

The author declares no conflicts of interest.
